# Variation in Tocochromanols Level and Mycotoxins Content in Sweet Maize Cultivars after Inoculation with *Fusarium verticillioides* and *F. proliferatum*

**DOI:** 10.3390/foods11182781

**Published:** 2022-09-09

**Authors:** Agnieszka Waśkiewicz, Małgorzata Muzolf-Panek, Łukasz Stępień, Elżbieta Czembor, Pascaline Aimee Uwineza, Paweł Górnaś, Marcin Bryła

**Affiliations:** 1 Department of Chemistry, Poznań University of Life Sciences, Wojska Polskiego 75, 60-625 Poznan, Poland; 2 Department of Food Quality and Safety Management, Poznań University of Life Sciences, Wojska Polskiego 31, 60-637 Poznan, Poland; 3 Department of Pathogen Genetics and Plant Resistance, Institute of Plant Genetics, Polish Academy of Sciences, Strzeszyńska 34, 60-479 Poznan, Poland; 4 Department of Biochemistry and Biotechnology, Plant Breeding and Acclimatization Institute—NRI, Radzików, 05-870 Błonie, Poland; 5 Institute of Horticulture, Graudu 1, LV-3701 Dobele, Latvia; 6 Department of Food Safety and Chemical Analysis, Prof. Waclaw Dabrowski Institute of Agricultural and Food Biotechnology—State Research Institute, 02-532 Warsaw, Poland

**Keywords:** *Fusarium*, sweet maize, resistance, mycotoxins, tocopherols, tocotrienols

## Abstract

A major problem in maize production is the contamination of the grain with *Fusarium* spp., mainly *F. proliferatum* and *F. verticillioides* and their secondary metabolites—mycotoxins. Under biotic stress conditions, caused by a fungal pathogen, plants initiate a series of defense mechanisms that may cause quantitative and qualitative changes in the composition of phenolic compounds. We analyzed the resistance of four sweet maize cultivars (Syngenta Group: Overland, Sweetstar, GSS 8529, Shinerock) to the infection with *Fusarium verticillioides* and *F. proliferatum* isolates, along with fumonisins B_1_, B_2_, and B_3_ grain contamination and the levels of tocopherols and tocotrienols accumulated. Differences in ear rot levels were found between the cultivars and isolates used. The phenotypic evaluation positively correlated with the concentrations of fumonisins. The results obtained also indicate a significant dependence on tocochromanols content in sweet maize cultivars tested on the infection of plants with *Fusarium* isolates and fumonisin biosynthesis. Further studies are needed to investigate the mechanisms of the plant reaction and the effect of different levels of tocopherols and tocotrienols on *Fusarium* resistance and grain contamination with mycotoxins.

## 1. Introduction

Maize (*Zea mays* L.)—one of the most important cereals—is a monoecious crop of the *Poaceae* family widely cultivated worldwide [1]. Along with wheat and rice it is the most frequently grown crop across the globe in both temperate and tropical regions with a cultivation area of around 200 million ha [2]. Maize is an essential and staple crop valued for its nutritional properties and used as food and animal feed, as well as raw material for industrial applications, such as biofuel and bioproducts [3].

One of the greatest challenges in maize production is the contamination of grain with *Fusarium* fungi and mycotoxins [4]. Rapid increase in maize cultivation area, use of inappropriate crop rotation and global warming of the climate have resulted in an increased incidence of diseases, including *Fusarium* ear rot caused mainly by *Fusarium proliferatum* and *F. verticillioides*, with yield reduction of 5–10% [3,5]. In addition, most *Fusarium* strains are able to produce mycotoxins during vegetation season [1,3,6,7,8]. As a result, mycotoxins reduce grain quality and, when consumed, cause various health problems for both humans and animals [3]. The most important secondary metabolites formed by *F. proliferatum* and *F. verticillioides* include group B fumonisins (fumonisin B_1_, B_2_, B_3_) [8,9]. The contamination of commercially available purified products for human consumption (ground maize grain, maize meal, grits, polenta, semolina, cornflakes and sweet maize) with these toxins typically does not exceed 1000 µg/kg, although it can be higher in some countries. The International Agency for Research on Cancer in 2002 classified fumonisin B_1_ under substances probably carcinogenic to humans (class 2B) [10]. Moreover, in 2007 the Regulation of the EC Commission no. 1126/2007 updated the highest admissible concentrations for the two most important fumonisins B_1_ and B_2_ found in maize and its processed products [11].

Breeding cultivars with increased resistance is commonly considered as the most profitable and environmentally friendly method of protection against *Fusarium* pathogens, since the use of fungicides is frequently not effective and largely dependent on weather conditions.

Constant progress in sweet maize breeding results in a considerable number of new cultivars of high economic importance, differing in vegetation period length, yield, contents of sugars and quality of kernels. Sweet maize is grown mostly for its nutritive and culinary value. The kernels contain all micro- and macroelements, as well as many vitamins: A, C, B1, B2, PP, and in particular large amounts of vitamin E [12]. Tocopherol and tocotrienols, together known as “tocochromanols”, are lipophilic compounds that are classified as tochochromanols [13]. However, according to Azzi (2019), the term ’vitamin E’ should not be generalized, being used interchangeably for different tocols, since only α-T meets the criteria of preventing the human deficiency disease of vitamin E ’Ataxia with Vitamin E Deficiency’ [14]. Tocochromanols are made of a chromanol ring and a polyprenyl side chain, which is saturated in tocopherols and three-fold unsaturated in tocotrienols. Both the tocopherols (Ts) and tocotrienols (T3 s) have four derivatives: alpha (α), beta (β), delta (δ) and gamma (γ), differing in number and location of methyl groups at the chromanol ring [12,15,16,17]. Particularly γ-T and α-T have the largest share of the total tocochromanols in maize [15]. In addition, kernels of sweet maize do not contain gluten, constituting a valuable dietary component in a gluten-free diet. Sweet maize provides fresh products for direct consumption and raw material for the fruit and vegetable processing industry, frozen food and preserves. Furthermore, numerous scientific studies have shown that consistent intake of whole grain maize reduces the risk of developing chronic diseases, such as cardiovascular disease, type 2 diabetes and obesity, and it also improves digestive health [18,19].

Under biotic stress conditions (e.g., caused by a fungal pathogen), plants initiate a series of defense mechanisms [20]. They also change quantitative and qualitative composition of phenolic compounds—plant secondary metabolites—participating in the regulation of pigmentation, growth, reproduction of plants and their resistance to pathogens [21]. Phenolic compounds may be divided into two groups: those synthesized during normal development and growth of plants, and those for which production is triggered by biotic and/or abiotic stress [22,23,24]. This process may cause inhibition or impaired development of the pathogen and/or limited biosynthesis of mycotoxins.

Knowledge about the impact of *Fusarium* infection on the levels of some secondary metabolites, e.g., tocopherols and tocotrienols in maize kernels, is still limited. Therefore, the objective of the present study was to investigate the effect of *Fusarium* infection on the level of mycotoxins and its correlation with the level of tocochromanols content in four sweet maize cultivars.

## 2. Materials and Methods

### 2.1. Plant Material

Four sweet maize cultivars (Syngenta Group: Overland, Sweetstar, GSS 8529, Shinerock) were used to evaluate the relationship between the levels of their resistance to the infection with two *Fusarium verticillioides* isolates and two *F. proliferatum* isolates, fumonisins B_1_, B_2_, and B_3_ grain contamination and the levels of tocochromanols accumulated in their kernels. Sweetstar represents an early genotype with an average of 14–16 kernel rows per cob. GSS 8529, Overland and Shinerock belong to an average 18 rows-per-cob group (as a mid-early or mid-late genotypes). They were included into this study based on the preliminary experiment as the susceptible or moderately resistant to ear rot under field condition.

### 2.2. Field Experiment and Phenotypic Ear Rot Resistance Assessment

Field experiment was conducted in 2016, in Radzików, Central Poland (52.2192′ N, 20.6315′ E, 87 m above sea level). An RCBD (randomized complete block design) model was used. About 25 plants were grown in one row, three replications (0.75 m between rows and 0.25 m between plants in the row). Four *Fusarium* sp. isolates were used: two *F. verticillioides* (KF 3492 and KF 3707) and two *F. proliferatum* (KF 3654 and KF 925). All isolates came from the KF collection of pathogenic fungi at the Institute of Plant Genetics, Polish Academy of Sciences, Poznań, Poland. Their species identities were confirmed using molecular techniques utilizing translation elongation factor 1α gene (*tef*-1α) sequence analysis, according to the previous works [7].

To produce inoculum, the isolates were grown on a liquid SNA medium. After 2 weeks, cultures were filtered through cheesecloth and conidial concentrations were adjusted to ~10^6^ spores/mL. Inoculation of individual ears was conducted 10–12 days after silking time using 1.5 mL of spore suspension. Minimum of 24 plants were inoculated for each genotype using a sterile needle (8–9 plants in three replicates). Control plants were inoculated using a sterile water without the pathogen. At maturity, ears from each plot were dehusked and harvested manually, dried to approximately 15% of grain moisture and individually rated for FER symptoms using a seven-point scale: 1 = no visible disease symptoms, 2 = 1–3%, 3 = 4–10%, 4 = 11–25%, 5 = 26–50%, 6 = 51–75%, and 7 = 76–100% of kernels exhibiting visual symptoms of infection, such as brown, pink or reddish discoloration of kernels (white mycelial growth) [25].

### 2.3. Tocochromanols Content

#### 2.3.1. Extraction of Tocochromanols

Extraction of tocochromanols was performed according to Górnaś et al. (2014) [26]. The amount of 0.1 g powdered maize kernels (5 maize cobs for each repetition) was placed in a 15 mL screw cap tube sequentially supplemented with 2.5 mL of ethanol, 0.05 g of pyrogallol and 0.25 mL of aqueous potassium hydroxide (600 g/L). The tube was immediately closed with a screw cap, vortexed for 10 s at 2500 rpm and incubated in a water bath at 80 °C for 25 min, during which (after 10 min) samples were vortexed for 10 s at 2500 rpm. After incubation samples were cooled immediately in an ice-water bath and 2.5 mL of sodium chloride (10 g/L) was added. The entire volume was vortexed for 5 s at 2500 rpm. Then, tocochromanols were extracted using 2.5 mL of *n*-hexane:ethyl acetate (9:1; *v*/*v*), and vortexed for 15 s at 2500 rpm and centrifuged for 5 min (1000× *g*, at 4 °C). The organic layer was removed to the round bottom flask and residues were re-extracted with 2.5 mL of *n*-hexane:ethyl acetate (9:1; *v*/*v*) as before. Re-extraction was repeated three times. The combined organic layer fractions were evaporated in a vacuum rotary evaporator at 40 °C to dryness, dissolved in 2 mL ethanol and filtered through a nylon syringe filter (0.22 μm).

#### 2.3.2. Determination of Tocochromanols by RP-HPLC/FLD

Tocochromanols were identified using a previously validated method [27]. The chromatographic separation was carried out on the Shimadzu HPLC system (Shimadzu, Kyoto, Japan) consisting of a pump (LC-10ADvp), a degasser (DGU-14A), a low pressure gradient unit (FCV-10ALvp), a system controller (SCL-10Avp), an auto injector (SIL-10AF), a column oven (CTO-10ASvp), a fluorescence detector (RF-10AXL), and Luna PFP column (3 μm, 150 mm × 4.6 mm) with a guard column (4 mm × 3 mm) (Phenomenex, Torrance, CA, USA). The analysis was performed under the following conditions: mobile phase methanol: water (93:7; *v*/*v*); flow (1.0 mL/min); column oven temperature (40 °C); room temperature (22 ± 1 °C) and runtime (13 min). The identification and quantification were performed using a fluorescence detector at an excitation wavelength of 295 nm and emission wavelength of 330 nm. The limits of detection (LODs) for tocopherols and tocotrienols were as follows: 0.051, 0.018, 0.022, 0.044, 0.061, 0.027, 0.030 and 0.019 mg/L for α-T, β-T, γ-T, δ-T, α-T3, β-T3, γ-T3 and δ-T3, respectively.

### 2.4. Mycotoxins Analysis

High purity fumonisin B_1_, B_2_, and B_3_ standards (50 µg/mL in acetonitrile: water, 1:1) LC/MS-grade organic solvents, water and other reagents were purchased from Sigma–Aldrich (Steinheim, Germany). Fumonisins were extracted and purified from 10 g of homogenized samples of maize kernels (5 maize cobs for each repetition) according to the detailed procedure described earlier [28]. The eluates were evaporated to dryness at 40 °C under a stream of nitrogen and stored at −20 °C until the chromatographic analyses. The analytical system consisted of the Aquity UPLC chromatograph (Waters, Manchester, MA, USA), coupled with an electrospray ionization triple quadrupole mass spectrometer (TQD) (Waters, Manchester, MA, USA). Chromatographic column—Waters ACQUITY UPLC HSS T3 (100 mm × 2.1 mm/ID, with a particle size of 1.8 µm) (Waters, Manchester, MA, USA) was used for mycotoxins separation, with a flow rate of 0.35 mL/min at room temperature. The mobile phase consisted of methanol (A) and water (B) with an addition of 0.1% formic acid, phase B additionally contained 2 mM ammonium formate. The gradient program was used: from 1% to 95% A in 10 min, then 95% A for 2 min, and return to initial conditions in 2 min. The injection volume was 3 µL. Mass spectrometer was operated in the positive electrospray ionization mode (ESI) with ion source/desolvation temperature 150/350 °C, respectively. The compounds were quantitatively analyzed using multiple reaction monitoring (MRM). The analytes were identified by comparing the retention times and m/z values obtained by MS and MS2 with the mass spectra (722.4/352.4, 706.4/336.4 and 706.4/170.4 for FB_1_, FB_2_ and FB_3_, respectively) of the corresponding standards tested under the same conditions. Limit of detection for fumonisins was 1 ng/g. All samples were analyzed in triplicate. For data processing Empower^TM^ 3 software was used (Waters, Manchester, UK).

### 2.5. Statistical Analysis

The statistical analysis was performed using Statistica 13.1 software (StatSoft, Tulsa, OK, USA). Hypotheses were tested at α = 0.05. The significance of the factors is presented as *p* value. Differences were considered significant at *p* < 0.05. Each combination of factors included 3 repetitions. Two-way analysis of variance (ANOVA) was performed (independent variables: maize species and pathogen isolate). The variance homogeneity was verified using Hartley–Cochran–Bartlett test. The HSD Tukey’s test was used to determine the significant differences between samples. Correlation between visual assesment and fumosisins and tocochromanols was tested using Spearman’s rank correlation coefficient (R). Principal component analysis (PCA) was used to visualize information and to detect relationship between variables. General discrimination analysis (GDA), the supervised pattern recognition method were used to calculate classification rules for samples discrimination. Cluster analysis (CA) as unsupervised pattern recognition method was also used to visualize data and show some patterns in dataset.

## 3. Results

Based on the obtained results it was possible to find the relationships between the levels of four sweet maize cultivars’ resistance to the infection with two *Fusarium verticillioides* and two *F. proliferatum* isolates, fumonisin B_1_, B_2_, and B_3_ content and the levels of tocochromanols accumulation in maize kernels. Statistically significant effects of both genotype (maize cultivar) and *Fusarium* isolate on fumonisins (FBs) content was observed based on two-way ANOVA.

### 3.1. Phenotypic Evaluation

Phenotypic evaluation of the cultivars’ resistance to ear rot caused by *Fusarium verticillioides* (KF 3492 and KF 3707 isolates) and *F. proliferatum* (KF 3654 and KF 925 isolates) after inoculation and under natural infection conditions was carried out at the maturity plant stage. Differences between Overland, Sweetstar, GSS 8529 and Shinerock cultivars were significant (*F* = 5.8843; *p* = 0.0015). Differences between ear rot levels after inoculation using each isolate or under natural infection were also found (*F* = 14.8081; *p* < 0.0001). On average, after inoculation the disease symptoms were scored as follows: 5.3 (KF 925), 5.5 (KF 3492) and 4.9 (both, KF 370 and KF 3654). Under natural infection it was scored as 2.2.

**Figure 1:**
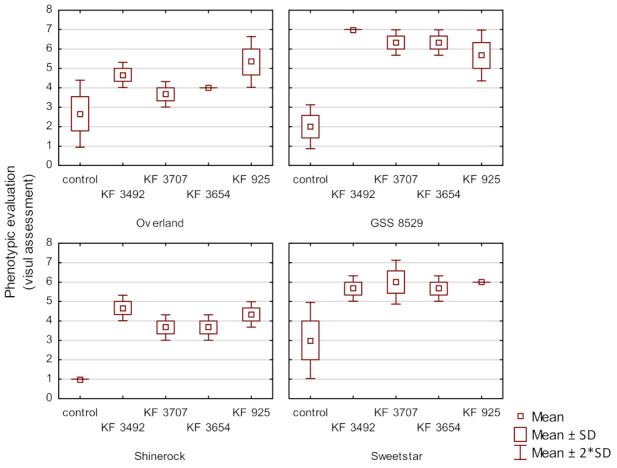
Mean values of ear rot resistance of four sweet maize cultivars (Overland, Sweetstar, GSS 8529 and Shinerock) inoculated with *Fusarium verticillioides* (KF 3492 and KF 3707 isolates) and *Fusarium proliferatum* (KF 3654 and KF 925 isolates) and under natural infection. Bars represent standard deviation (SD).

Sweetstar and GSS 8529 are very susceptible to ear rot (Figure 1). The level of resistance of the Sweetstar cultivar, which belongs to the early group with an average 14–16 rows per cob, was scored in the range from 5.7 (after inoculation with KF 925 isolate) to 7.0 (after inoculation with KF 3492 isolate). The GSS 8529 belongs to the mid-early group with an average 18 rows per cob and it was scored in the range from 5.7 (inoculation with KF 925 isolate) to 7.0 (after inoculation with KF 3492 isolate). Overland and Shinerock were described as moderately resistant to most of the isolates used. Both cultivars were described as mid-early and mid-late. Ear rot resistance of the Overland cultivar was scored in the range from 3.7 (after inoculation with KF 3707 isolate) to 5.3 (after inoculation with the most aggressive KF 925 isolate) and Shinerock from 3.7 to 4.7 (after inoculation KF 3492 isolate).

### 3.2. Mycotoxins Level

All results of mycotoxins concentrations were shown in Table 1. Two-way ANOVA revealed the statistically significant effects of both maize cultivar and *Fusarium* isolate on fumonisins (FBs) content. The highest values for all fumonisins (FB_1_, FB_2_ and FB_3_) were reported in GSS 8529 cultivar inoculated with *F. verticillioides* (KF 3707 isolate) and *Fusarium proliferatum* (KF 3654). Notably, the levels of all fumonisins were elevated in the samples of Overland cultivar inoculated with *Fusarium verticillioides* (KF 3707 isolate), and the samples of Overland and Sweetstar inoculated with *Fusarium proliferatum* (KF 3654 isolate).

### 3.3. Tocopherols and Tocotrienols Content

All results for tocochromanols content were shown in Table 2. Two-way ANOVA revealed the statistically significant effects of both maize cultivar and *Fusarium* isolate on tocopherols and tocotrienols content. The highest level of δ-T3 was observed in Overland and Sweet Star, γ-T3 in Overland and α-T3 in Shinerock cultivar (Table 2). The lowest levels of all three T3 s were observed for GSS 8529 cultivar, especially the samples inoculated with KF 3707 and KF 3654 *Fusarium* isolates. For tocopherols, the highest level of δ-T was reported in Sweetstar cultivar, β-T in Sweetstar and Overland, γ-T in Sweet Star and α-T in Sweetstar and Overland cultivars. Similarly, the lowest content of tocopherols was observed in GSS 8529 cultivar, especially in the samples inoculated with KF 3707 and KF 3654 *Fusarium* isolates. Generally, the maize cultivar could be ordered according to the decreasing content of total tocotrienols and tocopherols as follows: Overland > Sweetstar > Shinerock > GSS 8529. No β-tocotrienol was found in any of the samples analyzed.

The phenotypic evaluation (visual assessment) was positively correlated with the concentration of fumonisins, which was proven by statistically significant R-Spearman’s correlation coefficient, equalled to 0.51, 0.42 and 0.54 for FB_1_, FB_2_ and FB_3_, respectively. Additionally, the results of phenotypic evaluation (visual assessment) were significantly, inverserely correlated with α-T3 (R = 0.61, *p* = 0.000) and α-T (R = 0.55, *p* = 0.000) levels.

### 3.4. Multivariate Analysis

**Figure 2:**
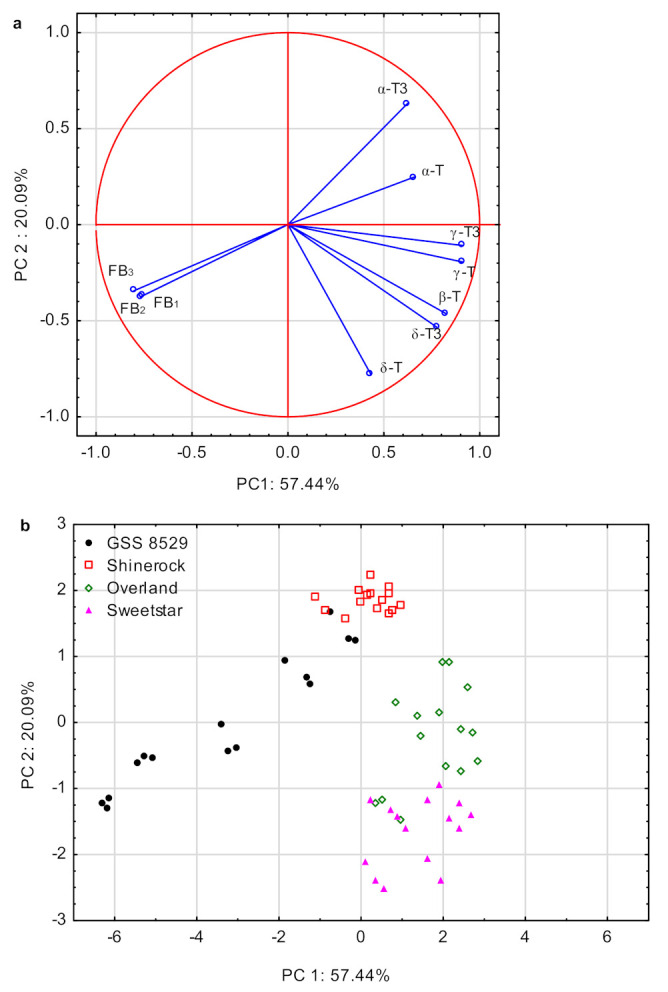
Projections of the variables (**a**) and scores (**b**) onto the factor plane defined by principal components (PC1 and PC2). Note: FB_1_, FB_2_, FB_3_—fumonisins B_1_, B_2_ and B_3_, respectively; α-T3, δ-T3, γ-T3—tocotrienols alfa, delta and gamma, respectively; α-T, β-T, δ-T, γ-T tocopherols, alfa, beta, delta and gamma, respectively.

In order to insert more insight into the data matrix and show the relationships between variables as well as variables and scores, PCA (principal component analysis) was performed. It could be stated that three principal components, showing eigenvalues higher than 1, explained around 92.5% of total variability, including the PC1 explaining 57.4% and PC2 explaining 20.1% of total variance. Figure 2 presents results of PCA, namely the distribution of variables (Figure 2a) and scores (Figure 2b) onto the factor plane described by PC1 as x axis and PC2 as y axis.

As shown in Figure 2a PC1 is highly positively correlated with γ-T3 (0.91), γ-T (0.91), β-T (0.82) and δ-T3 (0.78) and negatively correlated with fumonisins FB_1_ (−0.76), FB_2_ (−0.77) and FB_3_ (−0.80). PC2 is highly correlated with δ-T (−0.78). In Figure 2b, the distribution of scores is shown along the PC1 and PC2 axes. The scores were grouped into two distinct clusters. The first cluster included Overland cultivar (mid-late, phenotyped as a moderate resistant to ear rot based on the symptoms of the disease under field condition after inoculation) and Sweetstar cultivar. Both cultivars displayed high levels of tocopherols (δ-T3, δ-T, γ-T3, γ-T and β-T) and low level of the fumonisin content in comparison to the most susceptible GSS 8529 cultivar. The second cluster included GSS 8529 and Shinerock cultivars. Susceptible GSS 8529 accumulated high levels of fumonisins and Shinerock had high levels of alfa-tocopherols (α-T and α-T3). Shinerock was described as a moderately resistant to ear rot based on phenotypic evaluation and accumulated low levels of fumonisins. Moreover, within the susceptible GSS 8529 group the samples were highly scattered along both axes which indicated that GSS 8529 inoculated with various isolates differed significantly in tocopherol content and fumonisin concentration. The lowest differences between *Fusarium* isolates were noticed for Shinerock cultivar, phenotyped as the most resistant to ear rot based on the symptoms of the disease under field condition after inoculation. The PCA is less sensitive to the differentiation of all samples, thus GDA (general discriminant analysis) was performed to group the samples based on the similarities within clusters. GDA is a supervised pattern recognition technique which enables us to obtain discriminant functions, maximizing the ratio of between-class variance and minimize the ratio of within-class variance. The discriminant function was statistically significant (*p* < 0.05). The standardized canonical function coefficients are shown in Table 3.

**Figure 3:**
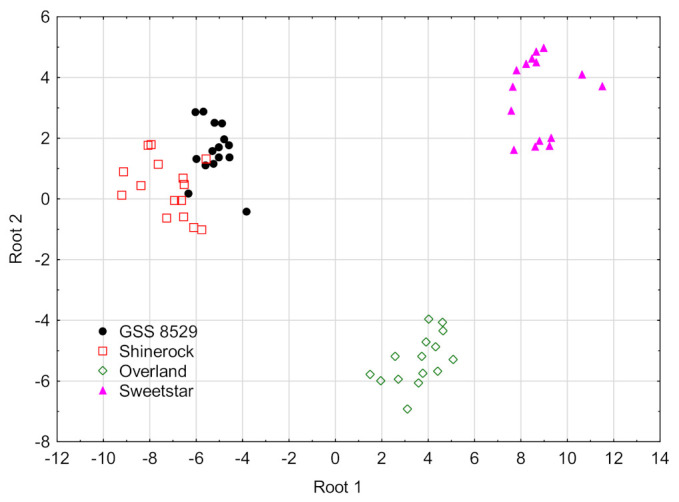
GDA classification of the scores (maize cultivars) based on tocopherols/tocotrienols and fumonisins contents.

Based on the results shown in Table 3 it could be concluded that the contents of α-T3, γ-T3 and FB_3_ contributed mostly to the first discriminant function which accounted for 78% of the total variance. The second canonical function explained 20% of total variance and was mainly related to δ-T3 content and FB_1_ and FB_2_ levels. Results of GDA discrimination was shown in Figure 3. All samples were discriminated with 97% classification propriety. Only the control samples of GSS 8529 cultivar were misclassified as Shinerock maize samples.

**Figure 4:**
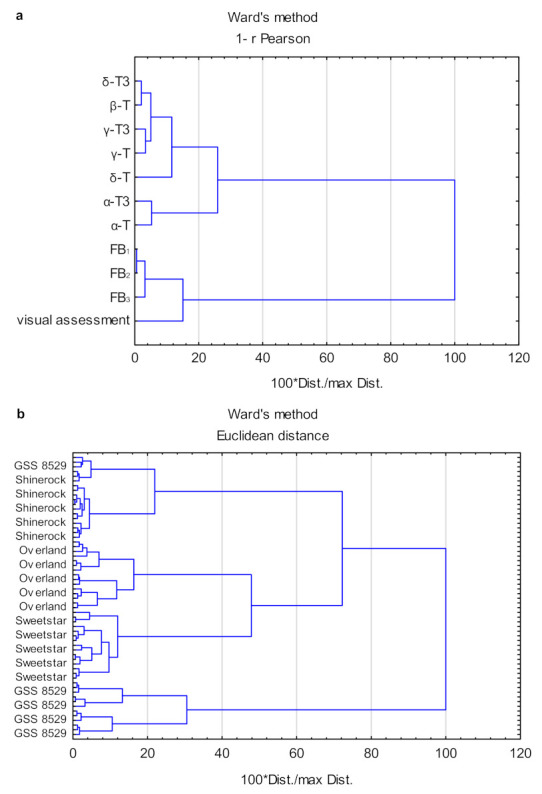
Dendrograms of variables (**a**) and scores–maize variety (**b**) of CA (cluster analysis) of similarities.

Next, the cluster analysis (CA) was performed to show the similarities between variables and similarities between samples. Results of CA was shown in Figure 4. Visual assessment was related to the fumonisin contents (the lowest 1 − r Pearson values). The highest 1 − r Pearson values were observed between results of visual assessment and α-T3 (1.50) and α-T (1.42) levels.

Figure 4b shows the dendrogram of the sample grouped according to their nearest similarities. At the 100 × Dist/max Dist. = 40 four distinct clusters could be observed. The first cluster included all Shinerock cultivar samples and three control samples of GSS 8529 cultivar, the second cluster consisted of all Overland cultivar samples, the third cluster of all Sweetstar cultivar samples and the fourth cluster included the GSS 8529 cultivar samples inoculated with *Fusarium* isolates. The results of CA were similar to the results of GDA, as not all GSS 8529 samples were properly grouped.

## 4. Discussion

The obtained results indicate a significant dependence of tocochromanols content in samples of sweet maize cultivars tested on the infection of plants with *Fusarium* isolates and mycotoxin biosynthesis.

This relationship can be clearly seen in the case of the susceptible cultivar GSS 8529 (especially after inoculation with KF 3707 and KF 3654 isolates), for which high concentrations of fumonisins (sum of FBs—21.58 and 19.85 µg/g, respectively) were shown. This finding corresponded to a significant decrease in the concentration of both tocopherols (9.14 and 10.35 mg/100 g, respectively) and tocotrienols (2.68 and 3.39 mg/100 g, respectively) compared to a control sample (1.24 µg/g—FBs; 18.13 mg/100 g—T; 6.60 mg/100 g—T3). The opposite situation was observed for the moderately resistant cultivar—Shinerock. Using each of the four isolates, the relation was similar—very low levels of fumonisins were noted (0.98–2.52µg/g for KF 3492 and KF 3654, respectively), which was associated with a significant increase in tocochromanols concentrations (Ts—21.27–23.44 mg/100 g and T3 s—7.72–8.30 mg/100 g) in the inoculated samples compared to the control (FBs—0.00 µg/g, Ts—17.20 mg/100 g; T3 s—4.75 mg/100 g). In the other two cultivars (Overland and Sweetstar), the situation was ambiguous and strictly dependent on the isolate. For the Overland cultivar, high concentrations of fumonisins were noticed only for variants with KF 3707 and KF 3654 isolates (12.15 and 6.84 µg/g, respectively), while the concentrations of tocochromanols remained at similar levels or even slightly higher (35.51 and 42.37 mg/100 g) compared to the control without inoculation (FBs—0.00 µg/g, sum of tocochromanols—35.03 mg/100 g). Other observations were made for the early Sweetstar cultivar, where, probably due to the shorter growing season, the level of fumonisins was low (1.07–1.24 µg/g), except for the variant with KF 3254 inoculation (7.68 µg/g), and the content of tocochromanols was slightly lower (29.25–34.94 mg/100 g) compared to the control (FBs—0.04 µg/g; sum of tocochromanols—37.01 mg/100 g).

There are only a few reports in the available literature, connecting maize fusariosis and mycotoxins biosynthesis with the level of various phenolic compounds. In the studies conducted by Martinez-Fraca et al. (2022) [29] the relationship between ferulic acid (FA) levels, fungal infection, and FB_1_ production was analyzed for 51 maize genotypes. Moreover, the putative antioxidative activity of ferulic acid—a major component of the seed pericarp—was verified. The authors selected maize cultivars with low and high FA levels and showed that fungal colonization (by *F. verticillioides*) and FB_1_ production were significantly lower in genotypes with high levels of FA than in genotypes with low content of FA [29]. Similar studies concerned the involvement of antioxidants (α-tocopherol, lutein, zeaxanthin, β-carotene and ferulic acid) in the resistance of maize cultivars to *Fusarium* ear rot mycotoxins accumulation [30]. It was shown that among the tested antioxidants, FA and α-tocopherol had the potential to inhibit the activity of *Fusarium verticillioides*, and their effectiveness depended on the susceptibility of the genotype. Majeed et al. (2017) [31] conducted research with the use of maize varieties with different susceptibility to fusariosis and analyzed the content of aflatoxins and tocopherols in these samples. The results showed that higher level of tocopherol corresponded with lower concentration of aflatoxins [31]. The presence of tocopherols and the aflatoxins content were also studied in another cereal—in different varieties of rice [16]. As in previous studies, the correlation between tocochromanols content and aflatoxins concentration was demonstrated—the higher the level of tocopherols in the samples, the lower the mycotoxin content.

Interesting studies were carried out on rats consuming maize naturally contaminated with mycotoxins, the diet of which was supplemented with selenium and vitamin E [32]. It has been shown that compared to the control group (without supplementation) selenium and vitamin E were able to alleviate oxidative stress and liver function damage due to the consumption of maize naturally contaminated with mycotoxins. In turn, Hoehler et al. (1996) [33] and Baldi et al. (2004) [34] investigated the effect of vitamins A, C and E on the toxicity of ochratoxin A and T-2 toxin in chickens and on cell lines, and showed that supplementation may, to a varying degree, depend on the type of antioxidant, counteracting a short-term mycotoxin toxicity.

Both our results and research conducted by other scientists show that there is still a lot to be explained and confirmed in this area.

## 5. Conclusions

Our results highlight the role of tocochromanols and their antioxidative activity on maize resistance to *Fusarium* ear rot and provide the basis of a phenotypic trait that can be deployed for breeding selection. The effect of *Fusarium* infection on the level of tocochromanols was particularly evident for the susceptible variety, where the high level of mycotoxins corresponded to a significant decrease in the concentration of tocochromanols. The results are interesting and need further studies to investigate the mechanisms of the plant reaction and the effect of different levels of tocochromanols on *Fusarium* resistance and grain contamination with mycotoxins.

## Figures and Tables

**Table 1:** Mycotoxin content [µg/g] in four maize cultivars inoculated with *Fusarium* isolates.

Maize Cultivar	*Fusarium* Isolate	FB_1_	FB_2_	FB_3_
	control	0.913 ± 0.033 ^f^	0.294 ± 0.033 ^gh^	0.031 ± 0.003 ^h^
	KF 3492	4.186 ± 0.340 ^d^	1.298 ± 0.151 ^de^	0.725 ± 0.071 ^b^
GSS 8529	KF 3707	16.061 ± 0.577 ^a^	4.553 ± 0.508 ^a^	0.969 ± 0.051 ^a^
	KF 3654	16.160 ± 0.286 ^a^	3.170 ± 0.303 ^b^	0.523 ± 0.050 ^c^
	KF 925	5.333 ± 0.178 ^c^	0.973 ± 0.160 ^ef^	0.137 ± 0.036 ^fg^
	control	0.000 ± 0.000 ^g^	0.000 ± 0.000 ^h^	0.000 ± 0.000 ^h^
	KF 3492	0.797 ± 0.084 ^f^	0.123 ± 0.021 ^gh^	0.057 ± 0.009 ^gh^
Shinerock	KF 3707	0.870 ± 0.060 ^f^	0.391 ± 0.013 ^gh^	0.019 ± 0.003 ^h^
	KF 3654	2.064 ± 0.174 ^e^	0.418 ± 0.028 ^gh^	0.033 ± 0.004 ^h^
	KF 925	0.996 ± 0.034 ^f^	0.217 ± 0.028 ^gh^	0.036 ± 0.008 ^h^
	control	0.000 ± 0.000 ^g^	0.000 ± 0.000 ^h^	0.000 ± 0.000 ^h^
	KF 3492	1.716 ± 0.174 ^e^	0.308 ± 0.030 ^gh^	0.049 ± 0.008 ^h^
Overland	KF 3707	9.562 ± 0.456 ^b^	2.171 ± 0.273 ^c^	0.417 ± 0.031 ^d^
	KF 3654	5.338 ± 0.237 ^c^	1.307 ± 0.262 ^de^	0.198 ± 0.014 ^ef^
	KF 925	2.229 ± 0.210 ^e^	0.576 ± 0.066 ^fg^	0.077 ± 0.014 ^gh^
	control	0.034 ± 0.006 ^g^	0.002 ± 0.000 ^h^	0.000 ± 0.000 ^h^
	KF 3492	0.863 ± 0.070 ^f^	0.152 ± 0.016 ^gh^	0.051 ± 0.010 ^h^
Sweetstar	KF 3707	1.012 ± 0.096 ^f^	0.221 ± 0.004 ^gh^	0.007 ± 0.001 ^h^
	KF 3654	5.960 ± 0.174 ^c^	1.493 ± 0.051 ^d^	0.229 ± 0.028 ^e^
	KF 925	0.917 ± 0.035 ^f^	0.128 ± 0.008 ^gh^	0.012 ± 0.004 ^h^

*n* = 3; KF 3492 and KF 3707—isolates of *Fusarium verticillioides*, KF 3654 and KF 925—isolates of *Fusarium proliferatum*; ^a–h^—the same superscript letters in the columns indicate the lack of significant differences between samples (α = 0.05).

**Table 2:** Tocopherols and tocotrienols content [mg/100 g of sample] in different maize cultivars inoculated with *Fusarium* isolates.

Maize Cultivar	*Fusarium* Isolate	α-T	β-T	γ-T	δ-T	α -T3	γ-T3	δ-T3	Total T	Total T3
	control	1.958 ± 0.202 ^fgh^	0.062 ± 0.018 ^cd^	9.269 ± 0.145 ^jk^	0.248 ± 0.044 ^cde^	2.011 ± 0.167 ^de^	4.209 ± 0.212 ^f^	0.377 ± 0.071 ^b^	18.133 ± 0.520	6.596 ± 0.437
	KF 3492	1.124 ± 0.045 ^jk^	0.048 ± 0.013 ^de^	8.097 ± 0.342 ^k^	0.297 ± 0.022 ^cde^	1.207 ± 0.068 ^hi^	2.611 ± 0.086 ^gh^	0.270 ± 0.042 ^b^	13.653 ± 0.589	4.087 ± 0.191
GSS 8529	KF 3707	1.668 ± 0.146 ^ghi^	0.007 ± 0.002 ^e^	4.592 ± 0.106 ^m^	0.200 ± 0.027 ^e^	0.920 ± 0.039 ^i^	1.496 ± 0.057 ^i^	0.263 ± 0.030 ^b^	9.144 ± 0.210	2.678 ± 0.125
	KF 3654	0.898 ± 0.061 ^k^	0.006 ± 0.002 ^e^	5.845 ± 0.263 ^l^	0.210 ± 0.021 ^e^	1.137 ± 0.090 ^hi^	2.026 ± 0.091 ^hi^	0.224 ± 0.051 ^b^	10.345 ± 0.502	3.386 ± 0.206
	KF 925	1.479 ± 0.098 ^ij^	0.060 ± 0.011 ^cd^	11.336 ± 0.239 ^fghi^	0.300 ± 0.043 ^cde^	1.816 ± 0.091 ^ef^	3.239 ± 0.141 ^g^	0.229 ± 0.047 ^b^	18.458 ± 0.643	5.284 ± 0.271
	control	1.954 ± 0.181 ^fgh^	0.062 ± 0.015 ^cd^	10.232 ± 0.396 ^ij^	0.204 ± 0.026 ^e^	1.801 ± 0.119 ^ef^	2.682 ± 0.198 ^gh^	0.261 ± 0.052 ^b^	17.196 ± 0.832	4.744 ± 0.365
	KF 3492	2.388 ± 0.128 ^cde^	0.050 ± 0.015 ^de^	12.246 ± 0.299 ^defg^	0.232 ± 0.033 ^de^	2.278 ± 0.173 ^bcd^	5.070 ± 0.144 ^e^	0.368 ± 0.065 ^b^	22.631 ± 0.822	7.716 ± 0.378
Shinerock	KF 3707	2.138 ± 0.066 ^def^	0.053 ± 0.009 ^de^	10.662 ± 0.310 ^hj^	0.206 ± 0.037 ^e^	2.493 ± 0.107 ^ab^	5.345 ± 0.163 ^e^	0.375 ± 0.017 ^b^	21.273 ± 0.700	8.213 ± 0.279
	KF 3654	2.518 ± 0.086 ^cd^	0.065 ± 0.015 ^cd^	12.349 ± 0.365 ^def^	0.215 ± 0.029 ^de^	2.507 ± 0.181 ^ab^	5.413 ± 0.221 ^e^	0.373 ± 0.047 ^b^	23.441 ± 0.884	8.294 ± 0.447
	KF 925	2.235 ± 0.069 ^def^	0.056 ± 0.012 ^cd^	11.767 ± 0.223 ^efgh^	0.214 ± 0.019 ^de^	2.704 ± 0.063 ^a^	5.217 ± 0.123 ^e^	0.375 ± 0.032 ^b^	22.569 ± 0.504	8.297 ± 0.204
	control	3.076 ± 0.091 ^ab^	0.144 ± 0.019 ^ab^	11.612 ± 0.231 ^efgh^	0.247 ± 0.017 ^cde^	2.237 ± 0.041 ^bcd^	7.139 ± 0.213 ^b^	0.600 ± 0.043 ^a^	25.057 ± 0.528	9.977 ± 0.283
	KF 3492	2.450 ± 0.092 ^cd^	0.119 ± 0.017 ^ab^	11.145 ± 0.323 ^ghi^	0.293 ± 0.018 ^cde^	1.810 ± 0.066 ^ef^	6.518 ± 0.182 ^bcd^	0.632 ± 0.058 ^a^	22.967 ± 0.706	8.960 ± 0.280
Overland	KF 3707	2.691 ± 0.171 ^bc^	0.132 ± 0.012 ^ab^	12.469 ± 0.319 ^def^	0.363 ± 0.034 ^bc^	2.138 ± 0.051 ^cd^	7.070 ± 0.164 ^b^	0.722 ± 0.043 ^a^	25.584 ± 0.785	9.929 ± 0.257
	KF 3654	3.366 ± 0.290 ^a^	0.124 ± 0.011 ^ab^	16.583 ± 0.549 ^a^	0.426 ± 0.025 ^b^	2.334 ± 0.108 ^bc^	7.879 ± 0.317 ^a^	0.724 ± 0.062 ^a^	31.435 ± 1.336	10.937 ± 0.485
	KF 925	3.298 ± 0.160 ^a^	0.140 ± 0.012 ^ab^	13.893 ± 0.435 ^c^	0.330 ± 0.024 ^bcd^	2.154 ± 0.090 ^cd^	7.015 ± 0.302 ^bc^	0.697 ± 0.078 ^a^	27.526 ± 1.091	9.865 ± 0.466
	control	2.031 ± 0.109 ^efg^	0.130 ± 0.017 ^ab^	16.748 ± 0.419 ^a^	0.655 ± 0.031 ^a^	1.733 ± 0.056 ^ef^	6.370 ± 0.259 ^cd^	0.621 ± 0.044 ^a^	28.288 ± 0.914	8.724 ± 0.359
	KF 3492	1.315 ± 0.138 ^ij^	0.102 ± 0.011 ^bc^	12.755 ± 0.700 ^cde^	0.685 ± 0.071 ^a^	1.400 ± 0.119 ^gh^	5.202 ± 0.422 ^e^	0.597 ± 0.097 ^a^	22.055 ± 1.534	7.198 ± 0.637
Sweetstar	KF 3707	1.621 ± 0.129 ^ghi^	0.153 ± 0.041 ^a^	13.200 ± 0.559 ^cd^	0.768 ± 0.081 ^a^	1.420 ± 0.052 ^gh^	5.110 ± 0.171 ^e^	0.626 ± 0.050 ^a^	22.898 ± 1.082	7.156 ± 0.272
	KF 3654	1.568 ± 0.049 ^hi^	0.128 ± 0.005 ^ab^	12.976 ± 0.408 ^cd^	0.759 ± 0.042 ^a^	1.590 ± 0.049 ^fg^	5.262 ± 0.177 ^e^	0.619 ± 0.038 ^a^	22.903 ± 0.764	7.472 ± 0.262
	KF 925	1.932 ± 0.107 ^fgh^	0.133 ± 0.004 ^ab^	15.219 ± 0.559 ^b^	0.656 ± 0.046 ^a^	1.696 ± 0.075 ^fg^	6.113 ± 0.296 ^d^	0.688 ± 0.064 ^a^	26.438 ± 1.151	8.498 ± 0.434

*n* = 3; control—maize not inoculated with *Fusarium*; KF 3492 and KF 3707—isolates of *Fusarium verticillioides*; KF 3654 and KF 925—isolates of *Fusarium proliferatum*; T—tocopherols; T3—tocotrienols; ^a–m^—the same superscript letters in the columns indicate the lack of significant differences between samples (α = 0.05).

**Table 3:** Standardized canonical discriminant function coefficients.

	Root 1	Root 2	Root 3
δ-T3	0.19	−0.38	−0.04
γ-T3	2.09	−0.6	0.23
α-T3	−2.51	0.55	−0.70
δ-T	0.56	0.95	−0.31
β-T	0.45	−0.64	0.37
γ-T	−0.13	0.10	0.07
α-T	−0.20	−0.42	−0.26
FB_1_	−0.24	−1.57	0.76
FB_2_	0.99	1.53	−0.52
FB_3_	−1.12	−0.49	0.46
discrimination %	78	20	2
cumulative %	78	98	100

## Data Availability

The data presented in this study are available on request from the corresponding author.
